# Rapid detection of *Bacillus* ionophore cereulide in food products

**DOI:** 10.1038/s41598-019-42167-0

**Published:** 2019-04-09

**Authors:** P. J. Ducrest, S. Pfammatter, D. Stephan, G. Vogel, P. Thibault, B. Schnyder

**Affiliations:** 1 University of Applied Sciences, HES-SO Valais//Wallis, Institute of Life Technologies, Sion, Switzerland; 2 University of Montreal, Institute for Research in Immunology and Cancer, Montreal, Quebec, Canada; 3 Mabritec AG, Riehen, Switzerland

## Abstract

Cereulide is a toxic cyclic depsipeptide produced by certain strains of *Bacillus cereus* found in soil and food products. While some harmless strains of *Bacillus* are used as probiotic, others can cause nausea and vomiting, and represent an important food safety concern. Current detection methods are time consuming and do not necessarily detect toxic cereulide. Here, we developed a rapid protocol using Matrix Assisted Laser Desorption/Ionization-Time of Flight (MALDI-TOF) mass spectrometry that detects the toxin originating from a colony smear of *B*. *cereus*. The distinct molecular feature of the toxin peak at m/z 1,191 was clearly identified from bacterial extracts with a limit of detection (LOD) of 30 ng/mL. Final optimisation of the sample preparation was based on cereulide chelating cations to produce the alkali adduct [M + K]^+^ without the use of a MALDI matrix, and provided a 1,000-fold improvement of LOD with 30 pg/mL of cereulide. We evaluated the application of this method for the detection of cereulide in rice, milk, and different ready-to-eat meals. The proposed protocol is quick, easy and provides an improvement over conventional methods for the detection of *B*. *cereus* toxin.

## Introduction

Contamination with soilborne *Bacillus cereus* is an emerging health safety concern for ready-to-eat foods due to their heat resistant endospores that can survive the cooking and manufacturing processes^1,2^. One third of *B*. *cereus* outbreaks originate from mixed food contaminations^3^. Heat-resistant endospores of some strains from *B*. *cereus* produce the toxin cereulide and can cause intoxications shortly after ingestion of contaminated food products, thus representing a serious health risk to consumers^4^. The occurrence of *B*. *cereus* in ready-to-eat food varies according to origins and manufacturing processes^5,6^ and 13.4% of foodborne outbreaks in inland China provinces between 1994 and 2005 were associated with this contaminant^7^. The prevalence and the health safety concerns related to food contaminated with *B*. *cereus* toxins require the development of a sensitive and rapid assay to detect this toxin.

Cereulide is a cyclic depsipeptide that contains three repeats of the amino acids (D-Oxy-Leu - D-Ala - L-Oxy-Val - L-Val)_3_ with a mass of 1,191 Da including the K^+^-adduct [M + K]^+^. Other variants of cereulide, termed isocereulide and homocereulide with different primary structures, and increased toxicity have been reported recently^8,9^. Emesis is usually recurrent as there is no vaccination against the toxin. The peptide is small and lacks immunogen properties, which partly explains the paucity of antibody-based cereulide tests on the market. In contrast, antibodies recognizing larger toxins such as those from *S*. *aureus* (26–28,000 Da) or from foodborne viruses are available, and can be used for protection of toxic shock and for toxin detection^10^.

Cereulide intoxication may exhibit severe or lethal symptoms in children and elderly people^11,12^. This toxin cause nausea, vomiting (emesis) and liver failure in children^13^. Vomiting hinders patients from seeking medical help, giving rise to a significant number of unregistered cases of foodborne intoxication. Furthermore, no causative agent is found in about one third of investigated foodborne outbreaks^14^. A number of these cases may have been caused by *Bacillus* and related toxins but they can only be confirmed when a routine test exists.

Cereulide can accumulate in various organs^15^ and lead to mitochondriotoxicity with clinical complications as hepatotoxicity, encephalopathy, and Beta cell dysfunction^13,16^. Improved management and monitoring of cereulide is required for appropriate clinical diagnostics. However, rapid and sensitive methods for the detection of cereulide are currently lacking, as screening methods are primarily identifying contaminating microorganisms from the *B*. *cereus* group without differentiating between pathogenic and non-pathogen strains. The *B*. *cereus* group includes the phylogenetically related species *B*. *cereus sensu stricto*, *B*. *anthracis*, *B*. *thuringiensis*, and a few less common members. However, the severity of the diseases associated with the corresponding pathogens differs remarkably, with *B*. *anthracis* causing fatal anthrax disease, *B*. *cereus* causing gastrointestinal disease with occasional severe symptoms, while others are non-pathogenic. A promising approach to differentiate between closely related species of *B*. *anthracis* and *B*. *cereus* has recently been reported using matrix-assisted laser desorption ionization- time-of-flight (MALDI-TOF)^17^. Seven putative biomarkers were identified in the protein spectra of *B*. *anthracis* which were not present in the other *Bacillus* species. Similarly, multiple putative biomarkers were identified in the MALDI-TOF mass spectra of emetic *B*. *cereus*^18^. While both studies examined protein biomarkers of molecular masses ranging from 2,000 to 12,000 Da, they did not report on the application of MALDI-TOF for the detection of lower molecular mass cereulide.

Today, the routine and official methods based on *ISO 7932* norms detect presumptive *B*. *cereus group* food contaminations, but do not detect cereulide which typically require time consuming human cell culture bioassays or extensive purification steps prior to mass spectrometry (MS) analysis^19,20^. In contrast, MALDI-TOF is a well-recognized and clinically certified method for routine identification of intracellular proteins of bacterial pathogens^21–24^. Here, we examined the analytical merits of MALDI-TOF for the detection of the extracellular cereulide in ready-to-eat meals.

## Results

### MALDI-TOF analysis of *Bacillus* toxin cereulide

**Figure 1:**
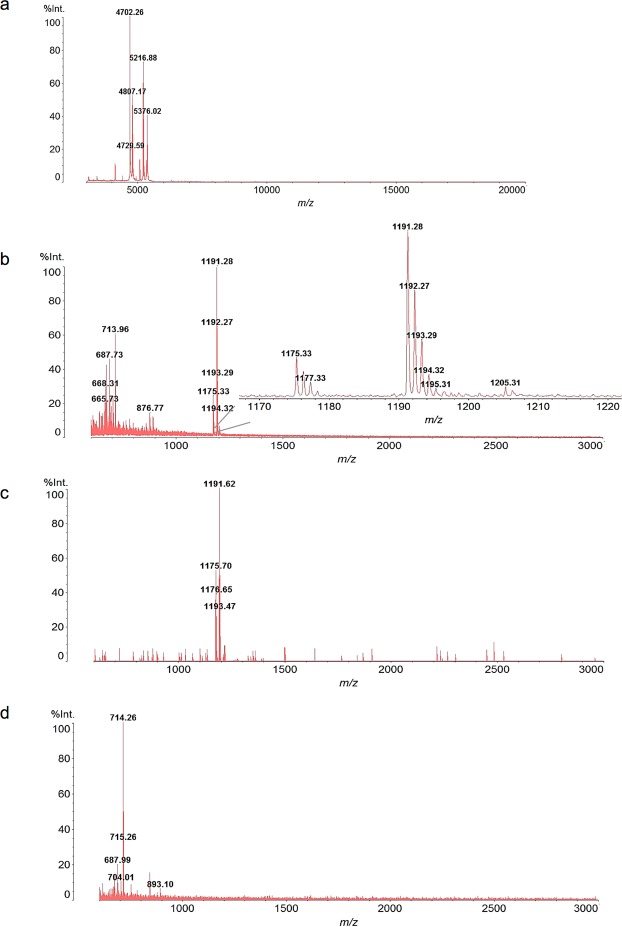
MALDI-TOF MS profiles of a culture colony smear of *B*. *cereus* strain *MB8* are shown, for the acquisition range (**a**) m/z 3,000–20,000 and (**b**) m/z 600–3,000. (**c**) Mass spectrum of the authentic cereulide-standard showing the [M + Na]^+^ and [M + K]^+^ ions at m/z 1,175 and 1,191, respectively. (**d**) Mass spectrum corresponding to the colony smear of the *B*. *cereus* reference-strain *DSM31* lacking cereulide. Results shown are representative spectra of at least three independent experiments.

A bacterial isolate of interest (*MB8*) was identified using MALDI-TOF. A colony-smear served as sample and a loop of sample was deposited on the MALDI plate for MS analysis (Fig. 1S). Sample preparation and subsequent MALDI-TOF analysis of this sample enabled the detection of distinct peaks with characteristic fingerprint (m/z 3,000–20,000 in Fig. 1a). The spectrum of protein masses was subsequently matched against reference spectra that are stored in the database and had initially been established using well characterized reference strains and defined growth conditions. Best match was achieved and the sample was reliably assigned to the *B*. *cereus* group. Dozens of additional *B*. *cereus* isolates were similarly identified, including strains *MB18*, *MB8/1*, *MB1*, *DSM31*, and further investigated in this study.

The same colony-smear of *B*. *cereus* (*MB8*) was also analyzed below m/z 3,000 resulting in the detection of peaks at m/z 1,175 and m/z 1,191 corresponding to the sodium and potassium alkali adducts of cereulide (Fig. 1b). The identity was confirmed using an authentic commercial standard of cereulide (Fig. 1c)^9,25^.

Two *B*. *cereus* isolates (*MB1*, *DSM31*) were selected, as these isolates lacked the cereulide synthetase genes^26^. MALDI-TOF MS analyses of the corresponding colony-smear samples confirmed the absence of cereulide peaks (Fig. 1d). The peaks at m/z 655 to m/z 844, possibly corresponding to lipids^27^ and were common to all *B*. *cereus* analyzed. Thus, the cereulide peaks at m/z 1,175 and 1,191 were only detected in *B*. *cereus* strains carrying the cereulide synthase genes *cesA*,*B* (e.g. strains *MB8*, *MB18*, *MB8/1*, *MB15*, *MB17*, *MB21*)^26^. These analyses indicated that under proper operating conditions MALDI-TOF can be used to identify *B. cereus* strains and differentiate between cereulide and non cereulide-producing strains.

**Figure 2:**
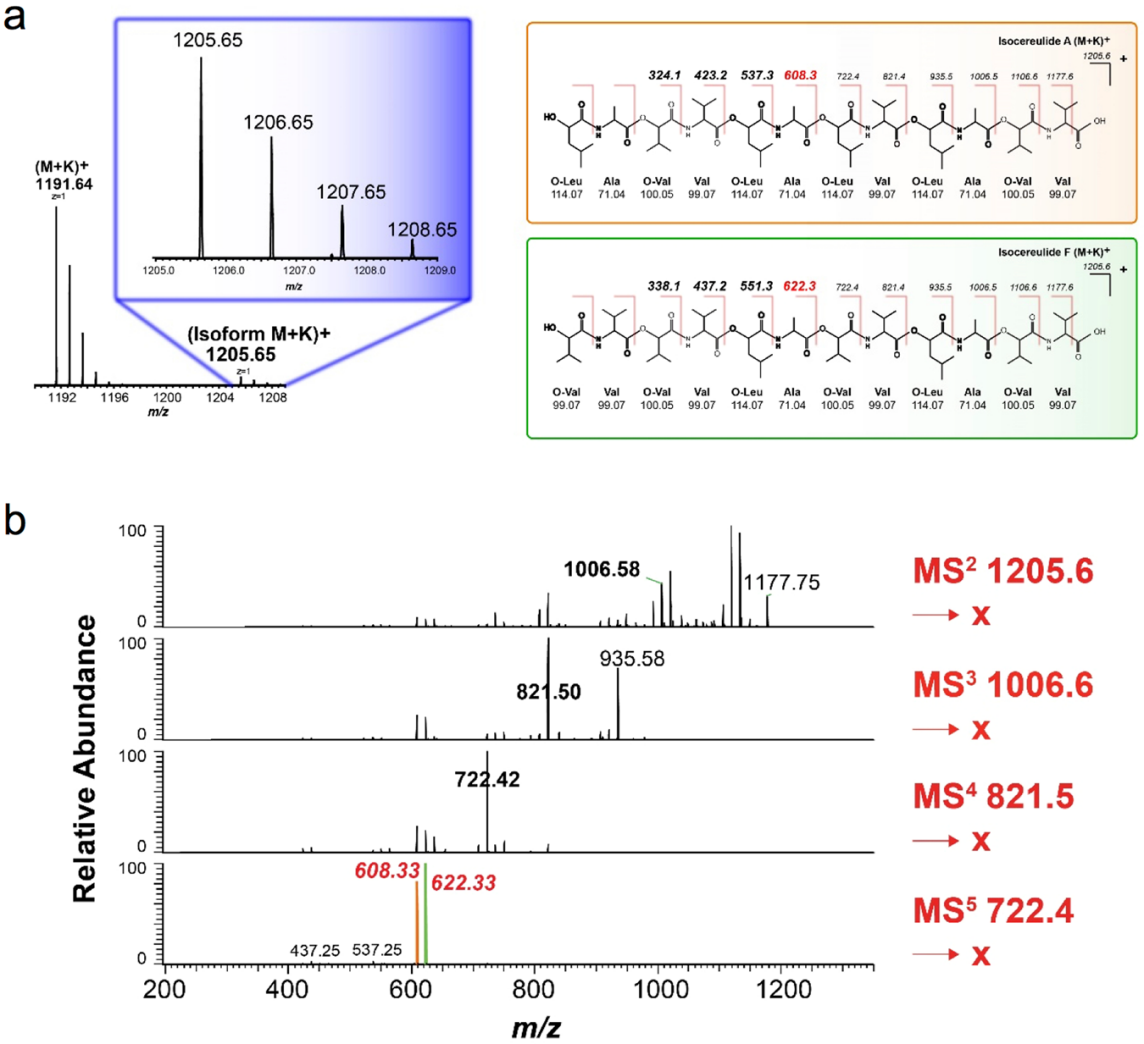
Identification of cereulide by MS/MS sequencing. (**a**) In the *B*. *cereus* cereulide preparation a peak at m/z 1,205.6 was observed. (**b**) Sequencing using the MS^n^ method identified the peak at m/z 1,205 as a mixture of iso-cereulides A and F.

As noted above, cereulide has an inherent propensity to bind alkali ions and this property plays an important role in dissipating the transmembrane potential in mitochondria of eukaryotic cells. The extent of alkali adduct observed in the MALDI-TOF mass spectrum depends on the buffer conditions in which cereulide is present. While the potassium adduct at m/z 1,191 was prominent in the MALDI-TOF mass spectrum shown in Fig. 1b, analyses performed using sodium buffers were dominated by the sodium adduct at m/z 1,175 (Fig. 2S). Furthermore, when pure cereulide was spiked into human urine the MALDI-TOF mass spectrum was dominated by a prominent [M + K]^+^ ion at m/z 1,191 peak, while spiked blood plasma analysis resulted in an abundant [M + Na]^+^ ion at m/z 1,175, reflecting the relative alkali concentration of the corresponding biological fluids.

### Sequencing of cereulide and a cereulide variant

The *B*. *cereus* peptide was sequenced by tandem mass spectrometry resulting in several fragment ions that can be used for confirmation purposes^9^ (Fig. 3S). The MS/MS spectrum of the [M + K]^+^ ion at m/z 1,191 resulted in the formation of fragment ions m/z 807.4 and m/z 423.2 corresponding to the loss of one and two (D-Oxy-Leu - D-Ala - L-Oxy-Val - L-Val) units, respectively. Additional fragment ions of high abundance such as m/z 608.3 corresponding to a loss of L-Oxy-Val - L-Val were also observed in Fig. 3S and can be used for further identification.

Interestingly, we noted that a peak at m/z 1,205 was frequently observed in the MALDI-TOF mass spectra of *B*. *cereus* extracts and was identified as a cereulide variant (Fig. 2). The identity of this variant was determined by multi-stage MS (MS^n^), where fragment ions from n^th^-generation product ions were selected in turn. These experiments enabled the identification of a mixture of cereulide variants including IsoCereulide A [(D-O-Leu-D-Ala-L-O-Val-L-Val)_2_(D-O-Leu-D-Ala-L-O-Leu-L-Val)] and IsoCereulide F [(D-O-Leu-D-Ala-L-O-Val-L-Val)_2_(L-O-Val-L-Val-L-O-Val-L-Val)] consistent with a recent study^9^. The two characteristic fragments for IsoCereulide A (m/z 608.3) and IsoCereulide F (m/z 622.3) appeared after MS^5^ selection of the ion product m/z 722.4. In conclusion, the peaks m/z 1,191 and m/z 1,205 were identified as cereulide and cereulide variant, respectively.

### Implementing the cereulide test in foodstuff

When applying the test cereulide was detected at the end of the exponential growth phase of *B*. *cereus* cultures at 13 °C and 40 °C. At 44 °C *B*. *cereus* grew but cereulide was not consistently detected. These results mirrored published findings using LC-MS analyses of cereulide^28–30^.

*B*. *cereus* inoculations into different food matrices at room temperature were then performed and cereulide production tested (Table 1). Inoculated 10 bacteria per gram of rice started the exponential growth after 4 h and cereulide was detected after 7 h. Foodborne cereulide was prepared by combining 1 g of food sample with 1 mL 75% (v/v) ethanol, by centrifugation and analysis of 1 μL of the supernatant. The same challenge test in milk, namely inoculation of 10 bacteria per milliliter, started the exponential growth after 7 h, and cereulide was detected at 15 h. The observed lower growth in milk, with maximally 1.6 × 10^8^ bacteria/mL milk, versus 1.8 × 10^9^ bacteria/g rice, explains the delayed cereulide detection in milk^31^. The lower oxygenation in milk correlates with lower metabolic activity and growth of the *B*. *cereus* germs compared to the higher growth on cooked rice grains.

**Table 1:** Food contamination with a cereulide producer germ.

*B*. *cereus* Inoculations	Detection of *B*. *cereus* exponential growth		Detection of cereulide			
	4 h	7 h	4 h	7 h	15 h	20 h
10 cells/g rice	+	+		+	+	+
10 cells/mL milk		+			+	+
10 spores/g rice		+			+	+
10 spores/mL milk		+				+

The symbol “+” in the table indicates the detection of exponential growth using plate counting, or of cereulide using MALDI-TOF MS at the indicated time points after *B*. *cereus* (MB8 strain) inoculation. Inoculation of foodstuff was performed with either vegetative cultures, named “cells” in the left column, or spores of *B*. *cereus*. Similar results were obtained for inoculation with 10 and 1000 cells/g. Saturation of the *B*. *cereus* growth curve in rice culminated in a maximum of 9.26 ± 0.14 (log_10_, cfu/g), for both, cells and spores inoculations, while growth culminated at 8.20 ± 0.04 (log_10_, cfu/mL) in milk. Results shown are representative for at least three experiments.

Then 10 endospores were inoculated per mL of milk or per gram of rice. Due to germination of the endospores, the resulting proliferating cells entering into exponential growth and their cereulide production were both detected although at later time (Table 1).

The cereulide-producer germ was further inoculated in all of the foodstuffs listed in Table 2, and the test applied. *B*. *cereus* cultures exponentially proliferated in meat-enriched foodstuff (minced meat, *Nasi goreng*), vegetable foodstuff (vegetable *Burger*), and starchy foodstuff (mashed potatoes, ricotta ravioli, milk-rice). Despite microbial growth, cereulide was not detected in the vegetable and meat-enriched foodstuffs, while cereulide was detected in the starchy food. To exclude a food matrix-effect interfering with the MALDI-TOF MS analysis of cereulide, we performed a spiking experiment. Pure cereulide was spiked into the vegetable and meat-enriched foodstuffs and successfully recovered by the test, indicating the absence of a food matrix-effect.

**Table 2:** Ready-to-eat food contamination with a germ capable to produce cereulide.

Ready-to-eat meals	*B*. *cereus*^*MB8*^ exponential Growth	Cereulide detection
**Meat-containing:**		
“Minced meat”	+	−
“Nasi Goreng”	+	−
**Vegetable-containing:**		
“Vegi Burger”	+	**−**
**Starch-containing:**		
“Milk-rice”	+	+
“Ricotta ravioli”	+	+
“Mashed potato”	+	+

Various ready-to-eat meals were inoculated with *B*. *cereus* (MB8 strain), as listed in the left column. The foodborne *B*. *cereus* cultures were analyzed for exponential growth (+), as indicated in the middle column. All inoculated food matrices were then analyzed by MALDI-TOF MS, and evaluated for the presence (+) or absence (**−**) of cereulide, as indicated in the right column. Results shown are representative for at least three independent experiments.

### Optimising the sample preparation

Changes in the sample preparation enabled improvement of the limit of detection value (LOD) of the test. A dilution series of standard cereulide was performed, in order to establish the LOD value. When the cereulide analysis was performed in presence of the same MALDI-matrix as for bacterial identification analyses^21^ the LOD was 30 ng/mL. The LOD of cereulide was lowered to 30 pg/mL when the MALDI-matrix was omitted. We termed the improved protocol LDI-TOF rather than MALDI-TOF mass spectrometry. Spontaneous chelation of the ionophore cereulide with K^+^-ions virtually contributes to this 1000-fold improved sensitivity of the test. Intriguingly, the presence of potassium in the sample matrix was sufficient for the production of prominent [M + K]^+^ ion without the requirement of a MALDI matrix, and significantly reduce the contribution of interfering matrix ions.

Implementation of the LDI-TOF MS cereulide test in foodstuffs confirmed the MALDI-TOF MS results shown above in Table 2. The lack in cereulide production in certain foodstuffs, for instance in the contaminated minced meat, was confirmed.

## Discussion

A protocol for the rapid and sensitive detection of cereulide in food was developed. Both, secreted cereulide and cytoplasmic proteins from *B*. *cereus* can be detected from the same colony-smear of *B*. *cereus* on agar culture plates. The acquisition of MALDI-TOF mass spectra below m/z 2,000 enabled the detection of potassiated cereulide ion at m/z 1,191 that was clearly distinguishable from the cytoplasmic protein peaks of *B*. *cereus*. Therefore, the cereulide method is compatible with existing MALDI-TOF methods used for pathogen identification, and thus provides a refinement thereof.

Cereulide can be detected by MALDI-TOF as [M + Na]^+^ and [M + K]^+^ adduct ions at m/z 1,175 and m/z 1,191, respectively. This ionophore binds *in vivo* to the negatively charged cell-membrane phospholipids and induces pore formation and cytotoxicity via leakage of K^+^ into the mitochondria^32^. Such molecular interaction is comparable to the cationic proteins of the cytoplasm which bind to negatively charged nucleic acids, via H^+^ or Zn^++^ cations^18^. Therefore, integration of K^+^ or Na^+^ adducts allows for the successful ionization by MALDI as well as LDI as shown for cereulide in the current study.

A final optimization is based on the native K^+^-chelation that favored the detection of cereulide by MALDI-TOF. No MALDI matrix was required in the sample preparation of cereulide. Background noise associated with matrix ions was minimized and enabled a 1,000-fold enhancement of LOD to 30 pg/mL. This LOD value was even several-fold lower than other LC-MS method for the analysis of cereulide (e.g. LOD: 100 pg/mL)^20^. The unique ionophore characteristic of this toxin facilitated the direct detection by LDI-TOF MS, while other microbial toxins tested in parallel (*S*. *aureus* enterotoxin^33^, *Cyanobacterium* microcystin) required the presence of the MALDI-matrix for successful detection.

The elaborated method detects cereulide (e.g. [M + Na]^+^: 1,175 and [M + K]^+^: 1,191) and cereulide variants (e.g. [M + Na]^+^: 1,189 and [M + K]^+^: 1,205), as well as the antibiotic valinomycin ([M + Na]^+^: 1,133 and [M + K]^+^: 1,149). These cyclic peptides have the property of forming K^+^/Na^+^-ionophore complexes and they share cytotoxic or anti-microbial activity. The principle of the method may be used for additional applications in the detection of the microbial toxin-*peptidome*. Here, the method enabled the rapid monitoring of emesis intoxications after food contamination with *B*. *cereus*. This method not only enabled the identification of *B*. *cereus* but also confirms the presence of cereulide in toxin strains. The test protocol identifies cereulide either bound to colony-smears from lab cultures or directly extracted from foodstuff, and can be implemented to address food safety concerns.

In challenge type tests, *B*. *cereus* producing cereulide was inoculated in ready-to-eat meals. *B*. *cereus* grew in all tested food matrices but only a selection actually produced cereulide. This toxin was detected in starchy foodstuff, and was not detected in vegetable and meat-enriched food matrices. These experiments confirmed the previous description of the non-ribosomal peptide synthases *ces* (NRPS) producing cereulide independently from ribosomal translation and cell growth^34^. In order to manage the risk associated with *B*. *cereus* strains producing cereulide the method described here can be used to screen suspected foodstuff for the presence of this toxin.

## Materials and Methods

### *B*. *cereus* cultures and analysis

*B*. *cereus* strains (including MB1, MB8, MB18, MB8/1, DSM31) were grown on plates of agar-containing medium (Trypticase soy agar (TSA); Biolife Italiana srl, Milano, Italy) at indicated temperatures. For routine enumeration of *B*. *cereus* colony-forming-units (cfu) by plate-counting, the selection media MYP (Mannitol Yolk Polymyxin) in agar-plates (Biolife Italiana srl, Milano, Italy) or the Columbia blood agar-plates (BioMérieux SA, Marcy l′Etoile, France) were used.

Colonies were further analyzed by MS using the smear method, where a colony was collected with a loop and deposited, in duplicates, on the MALDI’s stainless steel target-plate (Industrietechnik mab AG, Basel, Switzerland). Microbe identification by MALDI-TOF MS was run as described previously^13^. Briefly, *B*. *cereus* colony samples were applied on the MALDI stainless steel target plates using a sterile loop. One μL of matrix (α-cyano-4-hydroxycinnamic acid) was added and left to crystallize for 10 minutes. The analysis was run within 1 minute using an Autoflex III smartbeam MALDI-TOF mass spectrometer (Bruker Daltonics, Billerica, USA) in positive reflectron mode equipped with a 200 Hz Nd:YAG laser. Alternatively the MALDI-TOF mass spectrometer (*Shimadzu*) was used. The protein fingerprint was identified in a database according to SARAMIS program and strains were identified by a coverage of 98% or higher, when compared with database *B*. *cereus* group isolates.

### Preparation of *B*. *cereus* spores

For endospore formation, *B*. *cereus* strains were incubated on AK medium (Biolife Italiana, Milano, Italy) at 30 °C for 10 days. Spores were then collected using 5 mL of sterile water (4 °C) and remaining germs were eliminated by heating at 80 °C for 20 min. The spores were centrifuged at 6,000 × g for 10 min, resuspended in 5 mL of cold sterile water containing 1% (w/v) of lysozyme. Spores were stored at 4 °C until use in challenge tests in foodstuffs (described below). Enumeration of the spore-deriving germinating *B*. *cereus* in food matrices was done by plate-counting.

### MALDI-TOF MS test of cereulide

Sample preparation of cereulide was performed by the smear method, where a bacterial colony was spotted onto the MALDI target plate (also used for the bacteria identification described above).

However, sample preparation of foodborne cereulide was performed by combining 1 g of food sample with 1 mL 75% (v/v) ethanol (Alcosuisse, Bern, Switzerland) in 15 mL tubes (Falcon tubes). Tubes were vortexed for 1 min, and centrifuged at 6,000 × g for 10 min. 1 μL of the supernatant was spotted, in duplicates, onto the MALDI target plate.

The sample on the target plate was dried on air (room temperature). One μL of the MALDI matrix α-cyano-4-hydroxycinnamic acid (Sigma-Aldrich, St. Louis, MO) was added to each spot and allowed to crystalize at room temperature. Alternatively, the dried sample on the target plate was also analyzed without adding the MALDI matrix, and was referred to as LDI-TOF MS type analysis.

MALDI-TOF MS was run as described previously for microbe identification^13^ and the identical protocol was run for the cereulide analysis, using the following instrument settings. MS analysis of cereulide was performed using a Shimadzu equipment (Shimadzu Biotech, Kyoto, Japan) in positive reflectron mode in the range of 600–3,000 mass-to-charge (*m/z*), and an acceleration voltage of 20 kV on an AXIMA Confidence mass spectrometer, equipped with a 50-Hz nitrogen laser (pulse width: 20 ns, 337 nm). 100 profiles with 20 laser shots each were acquired automatically on 100 raster points separated by a distance of 120 μm. The pulsed extraction was set to 1.2 kDa and the laser power adjusted to obtain optimal signal intensities and resolution. External calibration of the instrument was performed using the “Peptide Calibration Standard II” mix from Bruker Daltonics, (Billerica, USA). Average profile spectra were collected from 20 laser shot cycles, and for every cycle 100 average profile spectra were stored. Spectrum analysis was conducted with the MALDI-TOF MS Launchpad 2.9 software (Shimadzu Biotech, Kyoto, Japan) with baseline corrections including peak filtering and smoothing. The resulting peaks were compared to effective mass-to-charge *m/z* values of standard cereulide (Chiralix B.V., Nijmegen, Netherlands)^17^.

Alternatively, MS analyses were also conducted on a Autoflex III smartbeam (Bruker Daltonics, Billerica, USA) in positive reflectron mode in the range of m/z 600–4,200 equipped with a 200-Hz modified Nd:YAG laser. Average profile spectra were collected from 10 cycles with 100 shots per cycle. Spectra analysis was conducted with the Flexcontrol Analysis version 3.0 (Build 185) with baseline correction and peak filtering and calibration with the “Peptide Calibration Standard II” mix (Bruker Daltonics, Billerica, USA) for low mass range peptides with a 500 ppm precision.

Limit of detection (LOD) value was calculated from a series of threefold and tenfold-dilutions of cereulide standard in 75% ethanol. LOD was defined as the lowest concentration of cereulide for which a signal-to-noise ratio of three was obtained using the MALDI-TOF MS instrument Bruker^35^.

### *B*. *cereus* challenge test in foodstuff

Fresh *B*. *cereus* cultures were grown overnight at 30 °C under shaking conditions (150 rpm) in complete broth medium TSB (Conda, Torrejon de Ardoz, Spain). They were added at the concentration of 5 × 10^4^ bacteria/g, to sterile closure bags (Seward, Worthing, UK) containing 200 g of the following foodstuffs obtained from a local retail store. Rice was cooked according to the instructions on the package (400 g of long-grain rice, added to 1 L of deionized water, was boiled for 19 min), cooled to room temperature and then inoculated with bacteria. UHT skimmed cow milk, minced meat (pork-beef), *Nasi Goreng* food, vegetable burger, ricotta ravioli, milk rice and reconstituted mashed potato powder were similarly inoculated with *B*. *cereus*. The inoculations were done manually, thoroughly homogenized, and the bags were incubated at 25 °C for indicated time periods. *B*. *cereus* counts were determined by aseptic removal of 20 g of food which were added to 180 g of a physiologic solution (containing 0.85% NaCl and 0.1% peptone (Biolife Italiana srl, Milano, Italy)) in stomacher bags (Seward, Worthing, UK), and by stomacher homogenization for 1 min. The homogenate was diluted in a decimal series and each dilution was spread onto TSA culture plates (Biokar Diagnostic, Beauvais, France). After 24 h at 30 °C, resulting colonies were counted and expressed as colony forming unit (CFU) per gram or mL of food. In parallel, the cereulide test was applied to the homogenized food samples prepared in the stomacher bag, as described above.

### Ionophoric property characterization of cereulide

A test was performed to confirm that the MS-peaks observed correspond to cereulide. One gram of *B*. *cereus* (strain MB8) culture-contaminated rice was weighted in 15 mL-Falcon tubes. Either 1 mL of a 1 M KCl solution or 1 mL of a 1 M NaCl solution was added, vortexed for 1 min, and incubated at room temperature for 15 min. After adding 8 mL of 100% ethanol, the foodborne cereulide was prepared as described in the cereulide test above and 1 μL of the supernatant was analyzed by MALDI-TOF MS.

### Amino acid sequencing of cereulide

Cereulide-containing extracts were sequenced using direct infusion nanoelectrospray-ionization tandem mass spectrometry (nESI MS/MS). The cereulide extract was diluted in methanol with 0.2% formic acid, and was analyzed at a concentration of 200 μg/μL on a LTQ-Orbitrap Elite (Thermo Fisher Scientific) with a flow rate of 600 nL/min in positive ion mode. The voltage of the spray was set to 1.6 kV and the source temperature was set to 320 °C. The full MS scan was acquired in the Orbitrap for the range m/z 150–2,000. For the MS/MS analysis, the precursor peak at m/z 1,191.64 was selected for fragmentation in HCD mode at normalized collision energy of 47%. This precursor peak at m/z 1,191.64 corresponds to [M + K]^+^ and was the most abundant ion. The MS/MS spectrum for the peptide sequence was assigned manually^36^.

## Conclusions

The presence of cereulide in foodstuff containing toxic *B*. *cereus* presents a serious safety concerns for the consumer. While several methods currently exist to detect bacterial contamination in food products, these cannot ascertain the presence of cereulide toxin. The MALDI-TOF developed as part of this study enables both the detection of bacterial proteins and the presence of cereulide in toxin-producing species, thus fulfilling an unmet need in the food industry. The confirmation of cereulide-positive *B*. *cereus* in the food process can launch immediate retrieval of contaminated food. This control guarantees toxin-free quality to address the increasing risk of *B*. *cereus* contaminated food, which now represents 1–10% of randomly sampled food products^5,6^. The implementation of this rapid and sensitive method would also contribute to important reduction of economic losses to the industry and improved food safety products on the market. Cereulide detection using MALDI-TOF is simple and sensitive, and can be implemented to ensure consumer protection and the quality of food processing.

## Supplementary information

Supplementary Dataset

## Data Availability

All data generated or analyzed during this study are included in the published article (and its supplementary information files).
