# Supplementary material for: Rapid detection of Bacillus ionophore cereulide in food products
Source: Sci Rep. 2019 Apr 9;9:5814. doi: 10.1038/s41598-019-42167-0 (PMC6456620; doi:10.1038/s41598-019-42167-0)
Supplement: Supplementary file 1 — Supplementary Dataset [file 41598_2019_42167_MOESM1_ESM.pdf]

Supplementary Information for

Rapid detection of *Bacillus* ionophore cereulide in food products

Ducrest PJ[1], Pfammatter S[2], Stephan D[1], Vogel G[3], Thibault P[2], and Schnyder B

1. Illustration of the MALDI-TOF MS test of a microbial sample using a colony smear

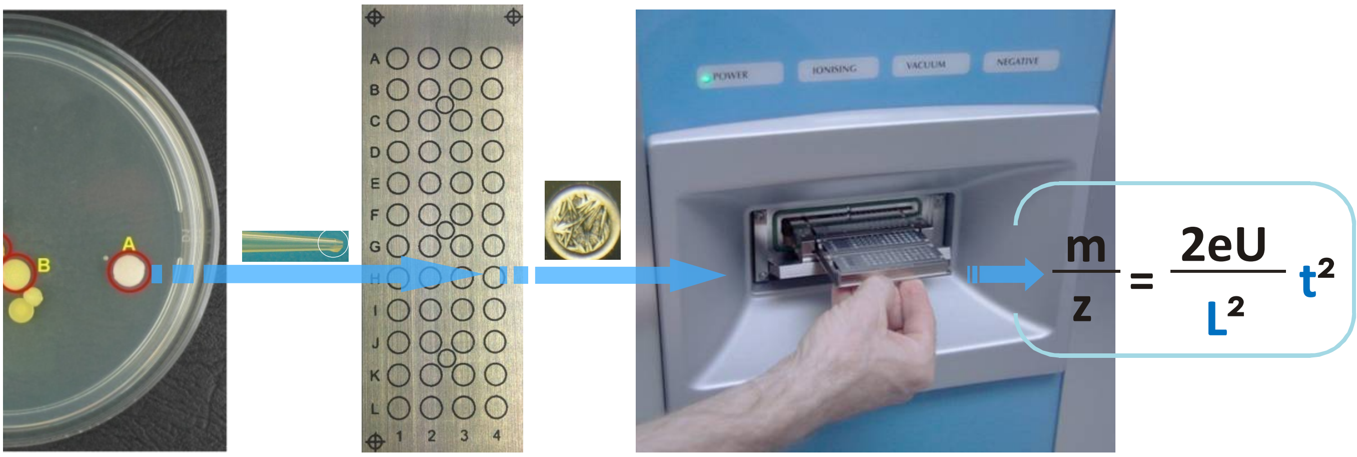

Figure 1S. Illustration of the MALDI-TOF MS test of a microbial sample using a colony smear. The sample was taken from an agar culture colony using a pipette tip (encircled) and transferred to the MALDI matrix on the target plate (central image). After drying / crystallization, the plate was inserted into the MALDI-TOF MS equipment. The test, including the transfer of identified m/z values, only takes a few minutes and can be run simultaneously for multiple samples. Used abbreviations stand for the following parameters, m: mass; z: charge; e: elementary charge; U: acceleration voltage; L: length of flight; t: time of flight (TOF).

2. Detection of cereulide in Potassium or Sodium riche matrixes

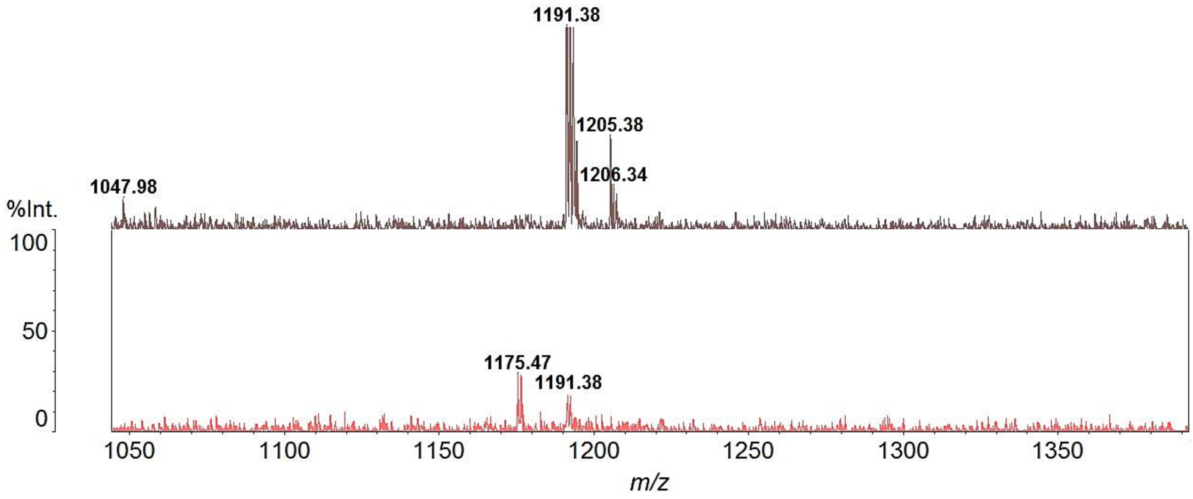

Figure 2S. *B. cereus* (*MB8* strain) was cultured in cooked rice and the contaminated food sample was either mixed with a 1 M KCl solution (upper panel) or with a 1 M NaCl solution (lower panel). The peptides of interest were extracted using ethanol and analyzed by MALDI TOF MS focusing on the profile range around the double peak at m/z 1,191 and 1,175, respectively, $[M+K]^+$ and $[M+Na]^+$.

3. Identification of cereulide

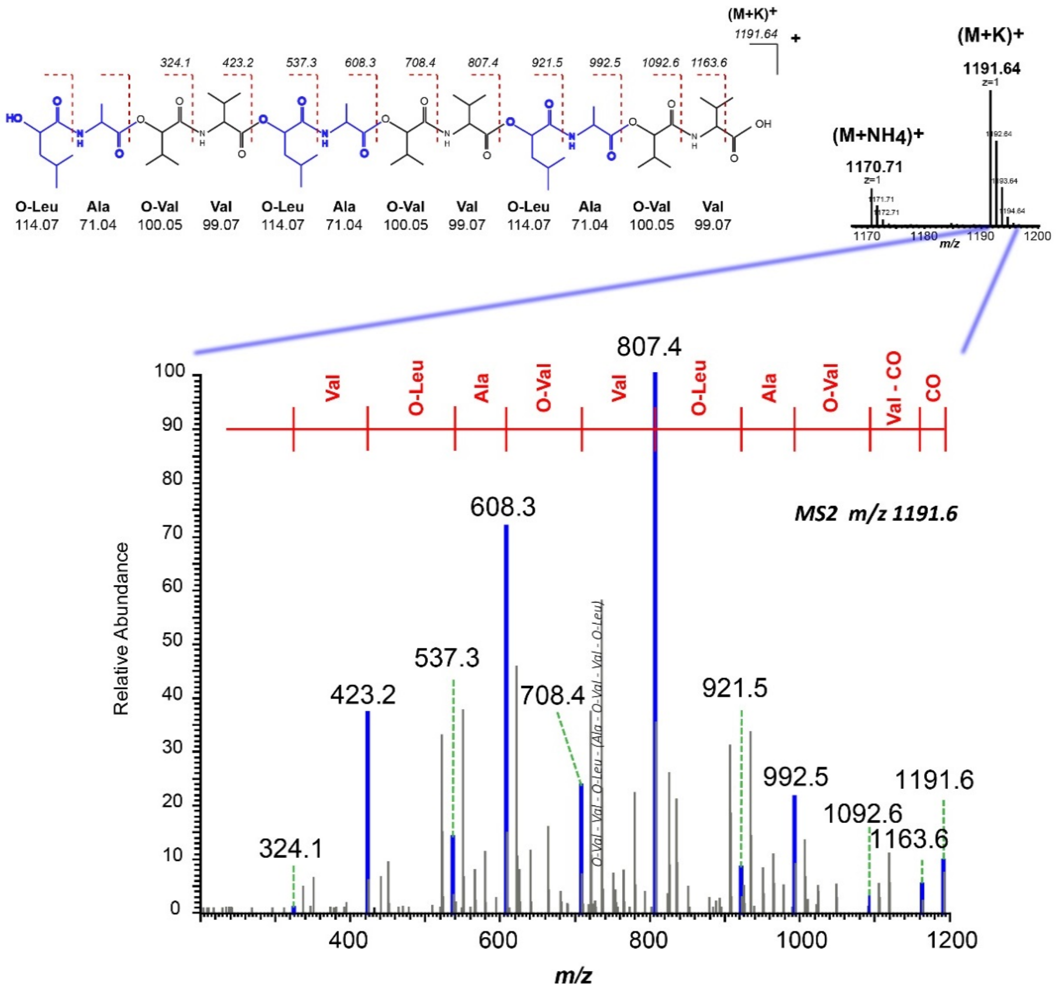

Figure 3S. Assigned MSMS spectra for the identification of cereulide $[M+K]^+$ m/z 1,191.6. Sequencing using the MS^n method identifies cereulide.
